# A rare case report of obstructive jaundice caused by mucus-producing cholangiocarcinoma

**DOI:** 10.1097/MD.0000000000028478

**Published:** 2022-01-21

**Authors:** Botao Duan, Xuekai Zhao, Songsong Fan, Lei Zhou, Xingyuan Zhang

**Affiliations:** Department of Hepatobiliary Surgery, Binzhou Medical University Hospital, Binzhou, China.

**Keywords:** case report, cholangiocarcinoma, inflammatory secretion, obstructive jaundice

## Abstract

**Rationale:**

Cholangiocarcinoma is a common cause of obstructive jaundice but is mainly associated with solid mass and not semisolid secretion. In this report, the patient was admitted to the hospital with obstructive jaundice; however, no solid mass was found to lead to jaundice.

**Patient concerns:**

The patient developed symptoms of obstructive jaundice for 10 days, including fatigue and yellow skin staining.

**Diagnoses:**

Postoperative pathological examination of the bile duct wall revealed cholangioadenocarcinoma, and the jelly like contents were inflammatory secretions.

**Interventions:**

The patient underwent laparotomy and was diagnosed with obstructive jaundice. An exploratory laparotomy revealed that the content in the biliary duct tree was a jelly like inflammatory secretion.

**Outcomes:**

Follow-up data revealed that the levels of total bilirubin and aminotransferase were normal, and a computed tomography scan showed no tumor mass.

**Lessons:**

There are very few reports about obstructive jaundice caused by inflammatory secretion that almost filled up the biliary tree. Internal drainage of the cholestatic bile can be achieved through endoscopic retrograde cholangiopancreatograpy, or external drainage can be achieved through percutaneous transhepatic biliary drainage, which can relieve the symptoms of biliary obstruction and improve the patient's quality of life.

## 1 Introduction

The pathogenesis of obstructive jaundice ranges from malignant to benign. Malignant causes include cholangiocarcinoma and pancreatic adenocarcinoma, while benign obstructive jaundice mainly originates from choledocholithiasis and chronic pancreatitis.^[1]^ Many patients undergo major operations such as choledochotomy and biliojejunal anastomosis. Obstructive jaundice due to bile duct obstruction by inflammatory secretions has rarely been reported. Herein, we present a case of cholangiocarcinoma in which obstructive jaundice was caused by inflammatory jelly like contents.

## 2 Case presentation

A 66-year-old female visited our hospital due to body weakness and jaundice. The patient suffered from gradually worsening complaints of generalized body weakness, jaundice, and anorexia for the last 10 days. On examination, vital signs showed icteric sclera and pale conjunctiva, and physical examination did not reveal any other remarkable signs.

**Figure 1:**
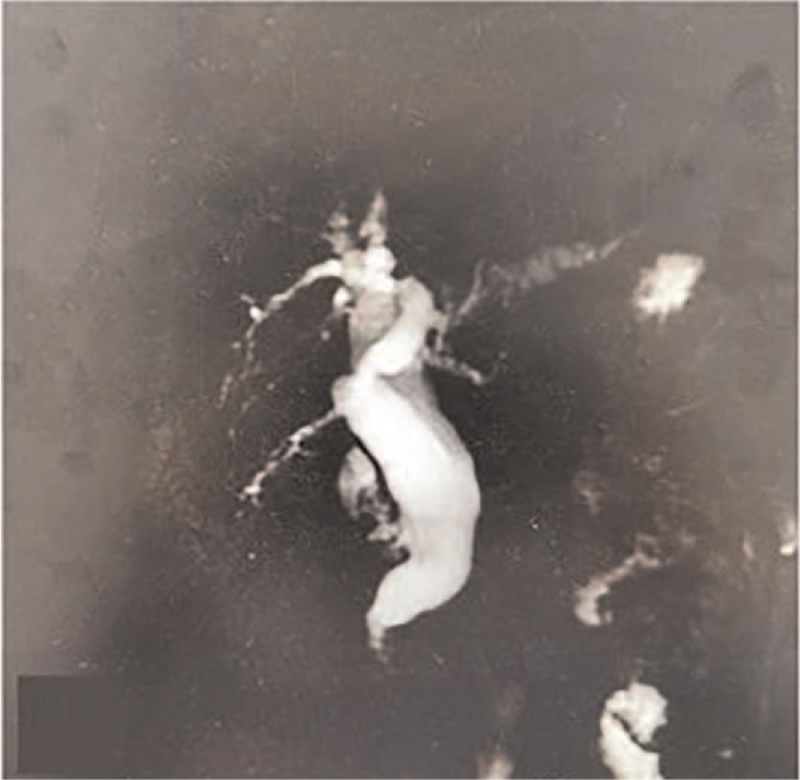
MRI showed extrahepatic ductal dilatation in the common bile duct region.

**Figure 2:**
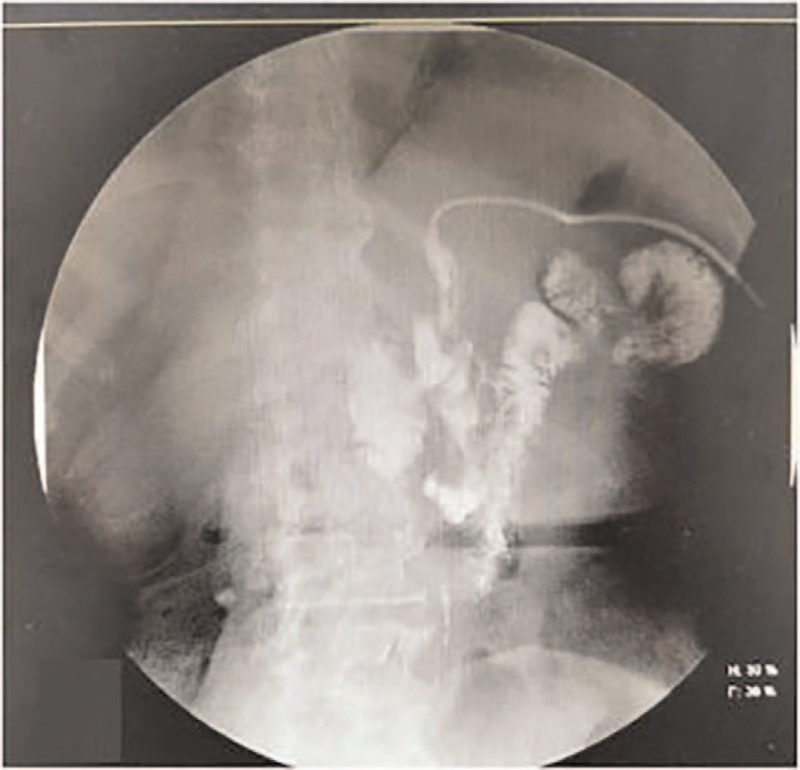
The percutaneous transhepatic cholangiography showed filling defect of bilateral intrahepatic biliary tree.

Laboratory data was significant for total bilirubin of 18.1 mg/dL (direct bilirubin 16.7 mg/dL, indirect bilirubin 1.4 mg/dL), alkaline phosphatase of 218 U/L, aspartate aminotransferase of 192 U/L, and alanine aminotransferase of 125.85 U/L. The serum tumor markers carcinoembryonic antigen level of 6.88 ng/mL, cancer antigen 19-9 level elevated over 1000 U/mL. Serology was negative for hepatitis B and C. Enhanced computed tomography scan and magnetic resonance imaging (MRI) revealed moderate intrahepatic and extrahepatic ductal dilatation measuring up to 2.0 cm in the common bile duct (CBD) region. No mass was found within or outside the liver; however there was an increase in the wall thickness at the end of the CBD (Fig. 1). A duodenoscope was performed to confirm the normal papilla and duodenum of the vater. To decrease the serum total bilirubin and improve liver function, ultrasound-guided percutaneous transhepatic cholangial drainage was performed. However, drainage of the bile was unsatisfactory. Percutaneous transhepatic cholangiography revealed a bilateral intrahepatic biliary tree filling defect, with the CBD filling poorly-distributed, while contrast could be directed downward to the Vater's papilla and into the duodenum (Fig. 2).

**Figure 3:**
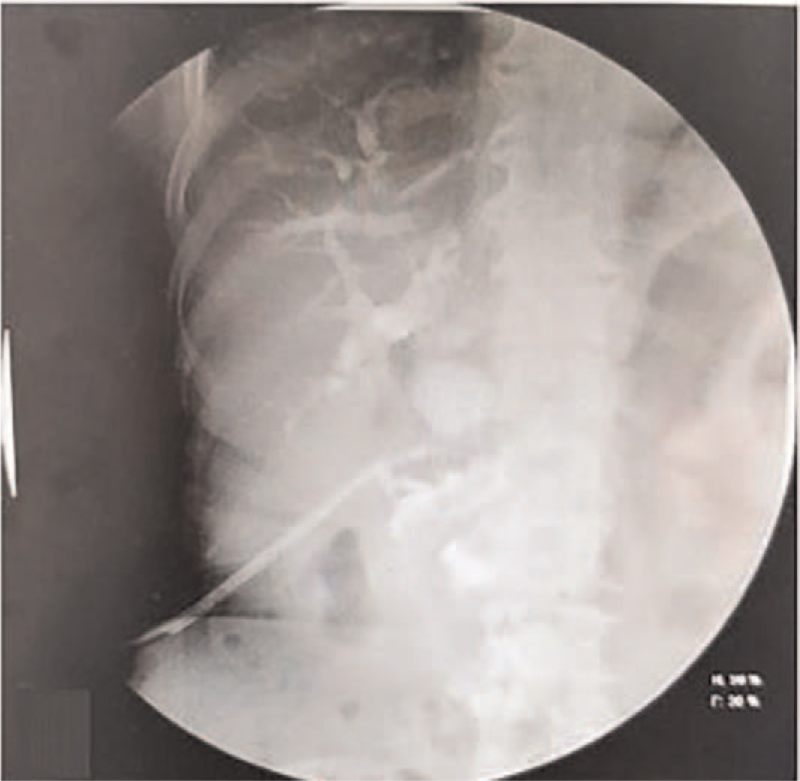
The percutaneous transhepatic cholangiography showed bilateral intrahepatic biliary tree filling better after the operation.

The patient underwent laparotomy and was diagnosed with obstructive jaundice. Open surgery revealed the following findings: moderate intrahepatic cholestasis, no mass in the abdominal viscera, and a CBD diameter of approximately 2.0 cm with local thickening. Surprisingly, many jelly like contents gushed at the point when the CBD opened (see Additional file 1). The intrahepatic and extrahepatic ducts were explored with a metal probe, which was directed upward into both hepatic ducts and passed downward through the papilla of the vater into the duodenum. A scoop was used to remove some jelly like contents, and a part of the CBD wall were sent for pathological examination. Choledochoscopy was used to further inspect the lumen of the duct, which revealed that the intrahepatic duct was almost filled with the secretion, which was difficult to wash out and could not be completely removed. After removing as much of the contents as possible, a No. 20 French double lumen T tube (the short arm was placed in the extrahepatic bile duct, the long arm was brought out through the abdominal wall, the inner tube was placed in the left secondary intrahepatic bile duct with the end outside the body connected to saline for flushing) was inserted, and a plastic catheter (diameter measuring about 0.2–0.3 cm) was placed in the right secondary intrahepatic bile duct for flushing (Fig. 3).

After the operation, bile flushing was carried out intermittently through both hepatic duct catheters, while approximately 150 to 200 ml/day bile drained from both tubes. Moreover, the patient serum total bilirubin level gradually became normal. Postoperative pathological examination of the bile duct wall revealed a cholangiocarcinoma with inflammatory cell infiltration. Jelly like contents was inflammatory secretions.

We suggested that the patient undergo radical resection of cholangiocarcinoma 1 month later; however, she refused due to economic problems. Recently, follow-up data revealed that the levels of total bilirubin and aminotransferase were normal, and a CT scan showed no tumor mass.

## 3 Discussion

When encountering a patient with obstructive jaundice, most surgeons would consider biliary stones or tumors as pathogenies.^[2]^ Cholangiocarcinoma is one of the main causes of biliary obstruction^[2]^ and was diagnosed in this case.

As a common malignant disease of the biliary tree, cholangiocarcinoma has an increasing incidence and has ranked as the first biliary malignancy in recent years.^[3]^ Sometimes, cholangiocarcinoma can have common symptoms such as weight loss, abdominal pain, emesis, and loss of appetite; moreover, when the mass of cancer obstructs the bile duct, the subsequent cholestasis changes many serological indexes, including total bilirubin, alkaline phosphatase, or transaminase, such as alanine aminotransferase and aspartate aminotransferase.^[3]^ As for the symptom, the patients with increasing volume of tumor mass may have painless jaundice.^[4]^ In the present case, the patient had elevated bilirubin, alkaline phosphatase, and transaminase levels. Moreover, she had symptoms of body weakness and jaundice. The tumor marker for the diagnosis of cholangiocarcinoma is Carbohydrate antigen 19-9 (CA 19-9), and this patient had very high serum cancer antigen 19-9. All above reasons indicated the probable diagnosis as cholangiocarcinoma.

Generally, most cholangiocarcinomas are at an advanced stage because many early cholangiocarcinomas have no markable manifestations.^[5]^ The patient was admitted to the hospital because of obstructive jaundice caused by mucus-producing cholangiocarcinoma. We found a few reports of obstructive jaundice caused by mucus-producing biliary duct tumors in the past ten years, although the mucus around the tumor does not fill the bile duct tree.^[6,7]^ Previous reports on intrahepatic cholangiocarcinoma and the operation scheme prioritize hepatectomy.^[8,9]^ However, the cancer was found in the extrahepatic bile duct wall in this case, whose physical fitness did not allow for radical surgery.

For the treatment of biliary obstruction caused by cholangiocarcinoma, biliary drainage and jejunal anastomosis are common options^[10]^; nevertheless, mucus may obstruct the bile duct again postoperatively. In this case, we combined a double-lumen T tube and a plastic catheter to innovatively drain both secondary intrahepatic vessels. After the patient's total bilirubin level became normal and liver function improved, radical surgery was considered.

In conclusion, for patients who have a clear diagnosis and are able to tolerate surgery, surgical operation can make the patients get better prognosis. The extent of resection depends on the location and the lesion area. For lesions confined to 1 segment, lobe, or half of the liver, hepatectomy can be performed to cover the entire lesion area. Extrahepatic bile duct resection and pancreaticoduodenectomy should be performed when the lesions involve the extrahepatic bile duct. As for patients with multiple diffuse lesions in both the left and right hepatic ducts, liver transplantation is often required when R0 cannot be achieved through liver resection. Literature reports that liver transplantation can achieve an ideal postoperative effect and low recurrence rate.^[11,12]^ If the patient is unable to undergo radical surgical resection or if his medical condition cannot tolerate surgical treatment, internal drainage of the cholestatic bile can be achieved through endoscopic retrograde cholangiopancreatography, or external drainage can be achieved through percutaneous transhepatic biliary drainage, which can relieve the symptoms of biliary obstruction and improve the patient's quality of life. There are very few reports about obstructive jaundice caused by inflammatory secretion that almost filled up the biliary tree, and we hope to provide a feasible approach for the treatment of such patients.

## Author contributions

**Methodology:** Xingyuan Zhang.

**Writing – original draft:** Bo Tao Duan, Xuekai Zhao, Songsong Fan, Lei Zhou.

**Writing – review & editing:** Bo tao Duan, Lei Zhou.
